# Isolation and Molecular Characterization of Two Lectins from Dwarf Elder (*Sambucus ebulus* L.) Blossoms Related to the *Sam* n1 Allergen

**DOI:** 10.3390/toxins5101767

**Published:** 2013-10-14

**Authors:** Pilar Jimenez, Patricia Cabrero, José E. Basterrechea, Jesús Tejero, Damian Cordoba-Diaz, Tomas Girbes

**Affiliations:** 1 Nutrition and Food Science, Faculty of Medicine and CINAD (Center for Research in Nutrition, Food and Dietetics; Lucia Building-Science Park), University of Valladolid, Valladolid E-47005, Spain; E-Mails: pilarj@bio.uva.es (P.J.); patricia_cabrero@hotmail.com (P.C.); joseezequiel.basterrechea@uva.es (J.E.B.); jesus.tejero@alumnos.uva.es (J.T.); 2 Pharmacy and Pharmaceutical Technology, Faculty of Pharmacy and IUFI (Institute of Industrial Pharmacy), Complutense University of Madrid, Madrid E-28040, Spain; E-Mail: damianco@farm.ucm.es

**Keywords:** *Sambucus ebulus*, blossoms, lectin, ebulin, ricin, ribosome-inactivating protein

## Abstract

*Sambucus* species contain a number of lectins with and without antiribosomal activity. Here, we show that dwarf elder (*Sambucus ebulus* L.) blossoms express two d-galactose-binding lectins that were isolated and purified by affinity chromatography and gel filtration. These proteins, which we named ebulin blo (A-B toxin) and SELblo (B-B lectin)—blo from blossoms—were subjected to molecular characterization and analysis by MALDI-TOF mass spectrometry and tryptic peptide fingerprinting. Both lectins share a high degree of amino acid sequence homology with *Sambucus* lectins related to the Sam n1 allergen. Ebulin blo, but not SELblo, was highly toxic by nasal instillation to mice. Overall, our results suggested that both lectins would belong to an allergen family exemplified by Sam n1 and could trigger allergy responses. Furthermore, they raise a concern about ebulin blo toxicity.

## 1. Introduction

Berries from *S. ebulus*, also known as dwarf elder, have been consumed since the Neolithic Age. In fact, it has been reported that berries from *S. ebulus* have been consumed as food or medicine since 5000 years ago in Italy and France [1,2]. To date, a number of compounds that display pharmacological activities, such as flavonoids, vitamins and lectins, have been isolated from *Sambucus* spp. [3,4,5,6,7]. Medicinal applications of *Sambucus* spp., like antiviral, anti-inflammatory and healing activities, have been described, and in some cases, the responsible molecules have been identified [8,9].

Lectins are one of the most studied *Sambucus* bioactive compounds [10]. Lectins (i) are proteins with sugar-binding ability and usually sugar specificity; (ii) some have cell-agglutinating activity; (iii) are ubiquitous in nature; and (iv) are found in all kinds of organisms [11]. In particular, they are very important in plant foods, since they may interact with the gastrointestinal mucosa, thereby changing the profile of bacterial interaction and may promote changes in glycoprotein expression on the mucosa surface that may result in changes in nutrient uptake [12]. A number of lectins with protein synthesis inhibitory activity, known as ebulins, have been isolated from different parts of *Sambucus ebulus*, like fruits (ebulin f), leaves (ebulin l) and rhizomes (ebulins r1 and r2) [13,14,15]. Ebulin toxins are known as type 2 (two chains) ribosome-inactivating proteins, or RIPs, since they have two chains, an A chain with *N*-glycosidase activity (E.C.3.2.2.22) on 28S rRNA, which is responsible for the irreversible inactivation of ribosomes, and a B chain with d-galactose-specific lectin activity [5,16,17]. *S. ebulus* also contains lectins of the B-B type with d-galactose-specific activity only, like SELld in leaves [17], SELlm in shoots [18] and SELfd in fruits [19,20]. 

Since ebulin shares amino acid sequences with *Sam* n1 isolated from blossoms, pollen and berries of *S. nigra* [21], we put forward the hypothesis that they and the related lectins devoid of translational inhibitory activity might be members of a broad family of structurally-related proteins (*Sambucus* lectins) of which *Sam* n1 allergen would be one of the family members. As a consequence, in a first approach to increase our knowledge on *Sam* allergens present in flowers that could trigger allergy and toxicity, we undertook studies aimed at investigating whether these kinds of lectins are present in dwarf elder blossoms. In the present research, we report two lectins structurally related to those found in fruits. We named them ebulin blo and SELblo. Ebulin blo was found to be a toxin of type A-B, highly toxic to mice by nasal instillation, while SELblo was found to be a lectin of type B-B devoid of acute toxicity comparable to ebulin blo. 

## 2. Results

### 2.1. Isolation of Dwarf Elder Blossom d-Galactose-Specific Lectins

**Figure 1:**
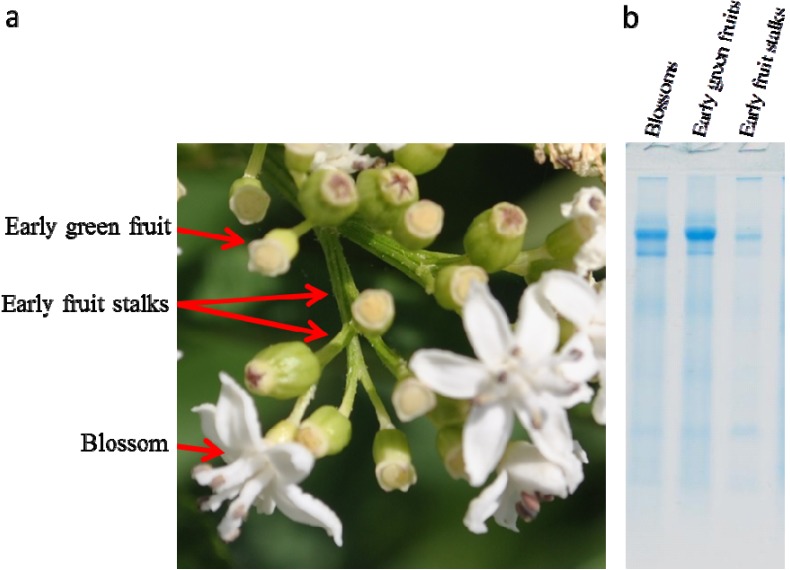
(**a**) Picture showing blossoms, early green fruits and early green stalks of *S. ebulus*. (**b**) SDS-PAGE of raw extracts of blossoms, early green fruits and early green stalks; 20 µL of extract concentrated by ultrafiltration were loaded into each well.

The SDS-PAGE of extracts prepared from dwarf elder blossoms revealed that they contain two major proteins in the zone of apparent Mr between 55,000 and 70,000 (Figure 1). These proteins were found also in early green fruits and early fruit stalks. We presumed that these proteins could be forms of the lectins previously reported in well-developed green fruits [16].

**Figure 2:**
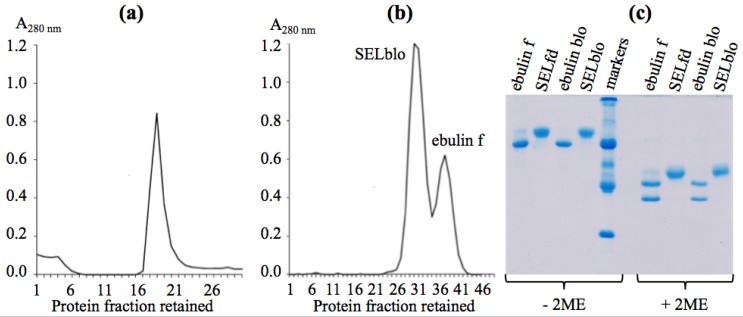
(**a**) Affinity chromatography of raw protein extract on acid-treated Sepharose 6B. (**b**) Chromatography of affinity-bound protein on Superdex 75. (**c**) SDS-PAGE of proteins purified from *S. ebulus* blossoms. The amounts of protein per well were ebulin f (11.5 µg), SELfd (11.1 µg), ebulin blo (9.5 µg) and SELblo (10.8 µg). The markers were, from top to bottom: SNAI (Mr 136 kDa), BSA (Mr 68 kDa), Ng b (Mr 58 kDa), ovalbumin (Mr 45 kDa), SNAIV (Mr 30 kDa) and trypsin inhibitor (Mr 20 kDa). 2-ME, 2-mercaptoethanol.

The isolation of these lectins was performed as reported recently to avoid the water-soluble mucilage that binds to the chromatography resin [19]. The procedure involves the first step of affinity chromatography on acid-treated Sepharose 6B, and the bound protein was eluted with lactose. That protein was subjected to a second chromatography on Superdex 75, which resolved two protein peaks, as seen in Figure 2. Fractions of the peaks were collected to avoid cross-contamination. The major peak accounted for SELblo and the other one for ebulin blo. The ratio of SELblo to ebulin blo in blossoms was higher than that reported for ebulin f in early and late green fruits [19].

### 2.2. Molecular Characterization by SDS-PAGE

Molecular characterization was performed first by SDS-PAGE in the absence or the presence of 2-mercaptoethanol (2-ME). As shown in Figure 2, both proteins, which we named ebulin blo (the smallest) and SELblo, moved as single and well-defined protein peaks in the absence of 2-ME, with apparent Mr close to 60,000 and 68,000, respectively. Both proteins were very close to those found in the extracts of green fruits [19]. In the presence of 2-ME, both proteins dissociated to yield two subunits of different apparent Mr, in the case of ebulin blo, presumably an A-type with an apparent Mr of 30,000, a B-type chain with an apparent Mr of 34,000 and, in the case of SELblo, two apparently identical B-type chains of 38,000. As control for electrophoresis, ebulin f and SELfd were run together with the lectins of blossoms. Both proteins agglutinated human red blood cells (O^+^), and that activity can be inhibited by these two sugars and was unaffected by d-glucose and d-mannose at concentrations up to 100 mM.

### 2.3. Molecular Characterization by Mass Spectrometry

**Figure 3:**
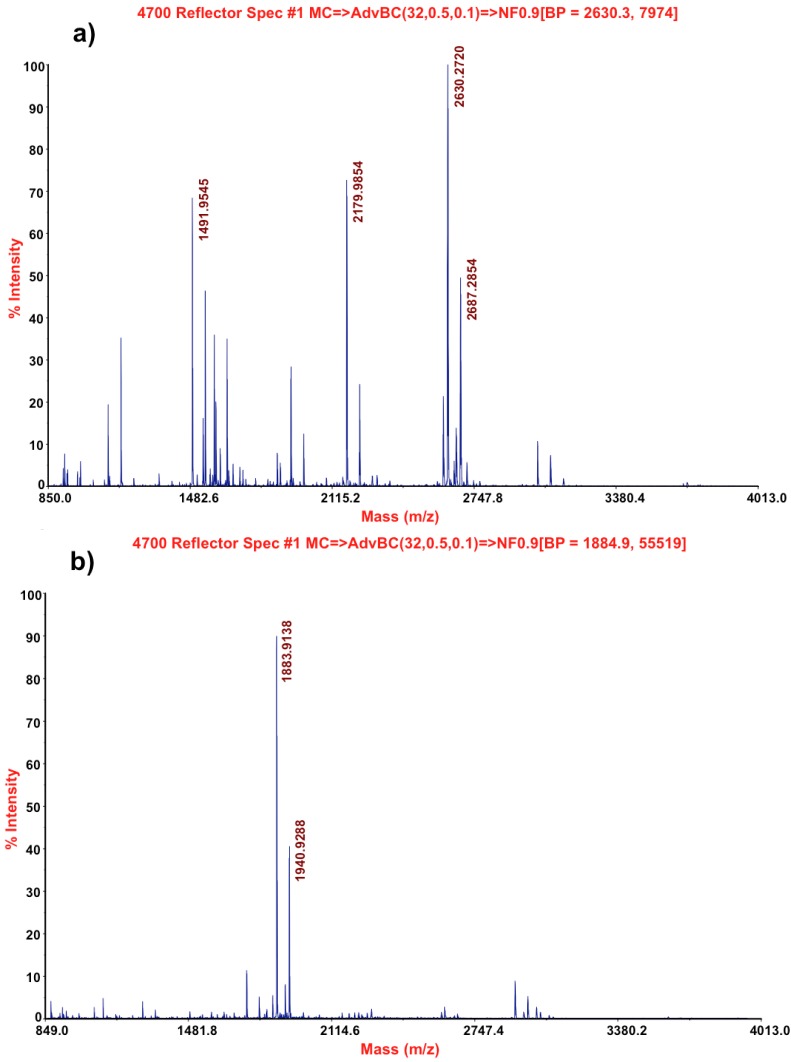
Mass spectrometry profiles of tryptic peptides from ebulin blo (**a**) and SELblo (**b**).

Mass spectrometry analysis of both lectins revealed values of mass 63,225 *m*/*z* and a calculated pI of 5.56 for ebulin blo, mass 68,432 *m*/*z* and a calculated pI of 5.54 for SELblo. In order to get the best identification of these proteins, tryptic peptides were prepared. As shown in Figure 3, both proteins yield different peptides, which suggests significant differences. Some of tryptic peptides from each lectin were sequenced by mass spectrometry.

**Figure 4:**
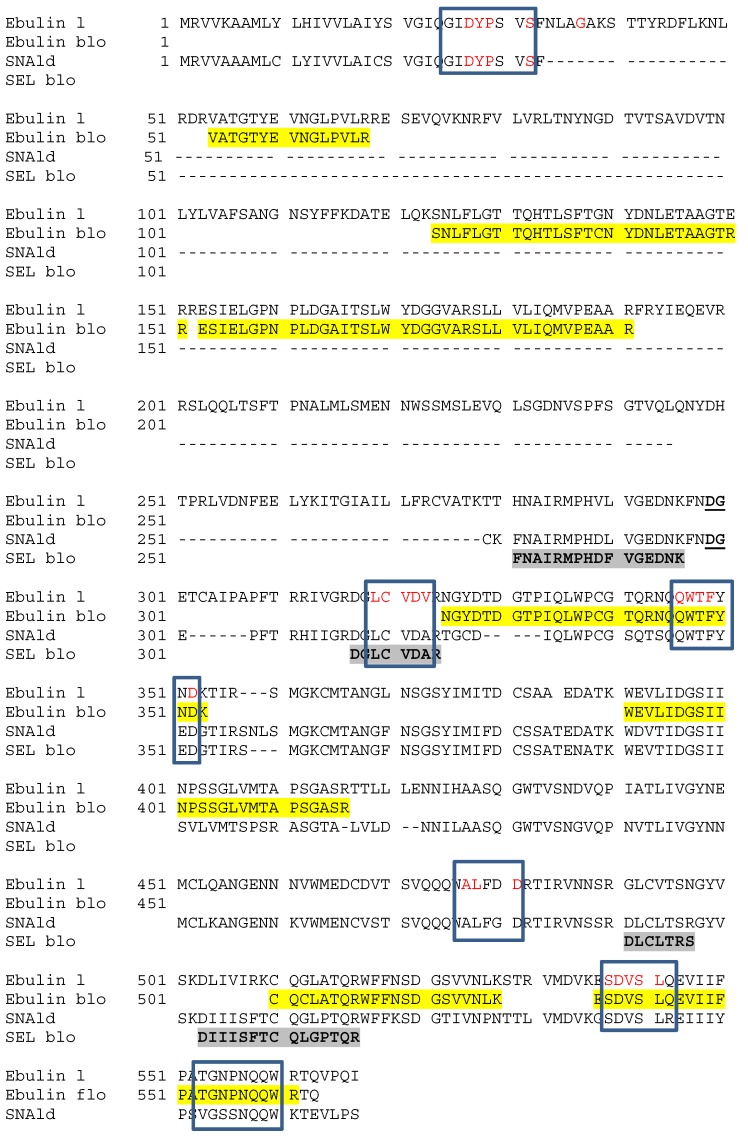
Alignment of amino acid sequences of some tryptic peptides of blossom lectins with ebulin l and SNAld. Boxed are sequences found in tryptic peptides from the allergen Sam n1 of *S. nigra* pollen. In red are coincident sequences. Highlighted in yellow and grey are tryptic peptides obtained from ebulin blo and SELblo, respectively.

Figure 4 shows the sequences of tryptic peptides from both blossom lectins aligned with the sequences of the ebulin l precursor from *S. ebulus* leaf (gi/13171073) and the SNAld precursor from *S. nigra* leaf (gi/32892180). Mascot search revealed that the sequences obtained from ebulin blo peptides fit with those of ebulin l in both the A and B chains ([14]; NCBI BLAST databank). Additionally, the sequence of SELblo peptides fit with those of SNAld. Furthermore, some peptides from the allergen, Sam n1, share a large amino acid sequence homology with the blossom lectins.

### 2.4. Toxicity of Dwarf Elder Blossom d-Galactose-Specific Lectins to Swiss Mice

**Figure 5:**
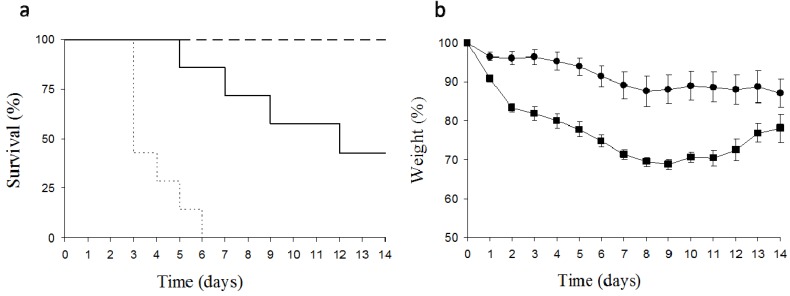
Effects of the instillation of ebulin blo in Swiss mice on the evolution of survival (**a**) and relative body weight loss (**b**). Dashed line and circles: instillation of 2.5 mg/kg body weight of ebulin blo; continuous line and squares: instillation of 5 mg/kg body weight of ebulin blo; dotted line: intraperitoneal administration of 5 mg/kg body weight of ebulin f.

The instillation of 5 mg/kg body weight of ebulin blo dissolved in water to both nasal cavities promoted a 30% reduction of body weight after nine days, which recovered afterwards (Figure 5). The reduction in weight was accompanied by the death of some animals five days after the treatment, reaching 50% mortality 14 days after application. Due to the clarity of the effect, the number of animals used was minimal. For comparative purposes, the effect of the intraperitoneal administration of 5 mg/kg body weight of ebulin f on the survival of mice has been included. 

The same test was performed with SELblo, the major protein isolated from blossoms of *S. ebulus*, and as could be expected, this B-b lectin did not cause toxicity in the assayed animals (data not shown).

The instillation of 2.5 mg/kg body weight of ebulin blo to female Swiss mice did not trigger deaths, but promoted toxicity revealed by a loss of weight, which reached 10% in seven days. This relative body weight loss was maintained until the recovery of the animals. Preliminary histological analysis indicates that ebulin administration orally promoted congestion in lungs (data not shown).

## 3. Discussion

Blossoms, early fruits and stalks contain lectins very similar to those present in fruits and in leaves [14,19]. By mass spectrometry, ebulin blo is slightly higher than ebulin f, while SELblo has the same Mr as SELfd. By SDS-PAGE analysis, both ebulin blo and ebulin f moved in gels with the same apparent Mr. The discrepancies in apparent Mr values determined by SDS-PAGE between the native protein and the summation of their subunits could be due to the different conformational states of the polypeptides when they are linked together in the native protein or when they are separated into their subunits. Ongoing experiments on intrinsic and aniline-naphthalene-sulfonate (ANS)-dependent fluorescence analysis indicated that ebulin f has a more flexible three-dimensional structure than SELfd, which facilitates locking-unlocking changes (data not shown). 

Both blossom lectins are d-galactose-binding proteins, which enables their isolation by affinity chromatography on acid-treated Sepharose 6B. The sequences of some tryptic peptides revealed a high amino acid sequence homology with the allergen, Sam n1, and other toxic and non-toxic *Sambucus* lectins [19].

According to our laboratory safety rules, we handled ebulin blo always in a water solution, since we suspected that ebulin blo powder would form aerosols and, therefore, could be highly toxic by inhalation, like ricin [22,23]. The administration of ebulin blo to mice was done under mild anesthesia to avoid undesired head and body movements and to ensure that all of the ebulin blo instilled was administered. Ebulin blo displays a dose-dependent powerful biological activity, as shown by its toxicity on the experimental animals when administered by nasal instillation. In this sense, a relative body weight loss between 20% and 30% was associated with animal death. After 4–5 days of the intraperitoneal administration of ebulin f, a related ebulin isolated from fruits, a relationship between body weight reduction and animal death (data not shown) was also found. The target for ebulin f in these conditions was the small intestine, which was very affected, and, in particular, the Lieberkühn crypts, which almost disappeared [24]. Oral ingestion of ebulin f was also highly toxic, but less than by intraperitoneal administration [18]. Therefore, the possibility that the toxic effects of ebulin blo triggered by nasal instillation would be through the swallowing of part of the ebulin blo instilled and transferring it to stomach, enough to trigger toxic effects, like those observed previously [19,24], cannot be ruled out. However, this is improbable, due to the concentrations used and the observed effects.

Some of the toxicity differences between ricin and ebulin would be related to the distinct intracellular traffic pathways followed by both proteins. Ricin has been shown to follow a retrograde transport from endosomes to the trans-Golgi network and, finally, transferring to the rough endoplasmic reticulum [25,26]. In contrast, ebulin, like nigrin, follows a pathway that goes from endosomes to lysosomes, where they are degraded and the inactive products expelled out of the cell [27,28]. The concentration-dependent accumulation of ebulin and nigrin in endosomes would favor the non-specific translocation to the cytosol and reaching the ribosomes. The unfolded protein response reported for ricin as an important factor contributing to its toxicity [29] should be also taken into account for ebulin.

Dwarf elder grows wild and abundantly in many temperate areas and requires no care. The isolation of lectins from any part of the plant even in raw form is relatively easy, due to its high concentration in the plant. Therefore, and as has been reported for ricin [30], from our point of view, ebulin blo at a rather high concentration can promote a serious toxicity nasally and orally and could also be considered as a bioweapon. Further work will approach the toxicity of raw preparations of these lectins, their potential neutralization by food matrices and liquids, the histologic effects in the main organs and the allergenicity of pollen, raw and purified lectins.

## 4. Experimental Section

All common chemicals and biochemical compounds were of the highest purity available, and most of them were purchased from Sigma-Aldrich (Sigma-Aldrich Química S.L., Tres Cantos, Madrid,). Sinapinic acid, trifluoroacetic acid and the α-cyano-4-hydroxy-transcinnamic acid matrix were purchased from Sigma-Aldrich. Isoflurane was purchased from Esteve Veterinaria (Barcelona, Spain). The chromatographic supports for protein isolation were purchased from GE Healthcare Europe GmbH (Barcelona, Spain). The affinity chromatographic support was acid-treated Sepharose 6B and was prepared by treatment of Sepharose 6B beads with 0.1 N HCl at 50 °C for 3 h and extensive washing with Helix^®^ Millipore water (Millipore Ibérica S.A.U., Madrid, Spain) [19]. Dwarf elder (*Sambucus ebulus* L.) blossoms were harvested from Barruelo del Valle (Valladolid, Spain) in early July and stored frozen at −20 °C until use. The previously prepared acrylamide (37.5%)-bisacrylamide (0.8%) mixture was obtained from Amresco (Solon Ind., Solon, OH, USA).

### 4.1. Isolation of d-Galactose-Binding Lectins from *S. ebulus* Blossoms

Two-hundred grams of frozen blossoms were minced and ground in a mortar to obtain a paste material, which was extracted overnight with 800 mL of extraction buffer (280 mM NaCl containing 5 mM sodium phosphate-pH 7.5). That raw extract was strained through cheesecloth, and the fluid was then centrifuged at 7500 g for 45 min at 4 °C, after which the supernatant was centrifuged again at the same speed for 30 min and removed and stored at 4 °C until use. The remaining plastic material with a mucilaginous consistency was removed by filtration through two layers of common laboratory filter paper. The filtered extracts were chromatographed through acid-treated Sepharose 6B (AT-Sepharose 6B) to obtain d-galactose-binding proteins, as reported previously for the lectins of dwarf elder fruits [16]. Seven-hundred and fifty milliliters of extract were applied to a XK50 (5 × 15 cm) column (GE Healthcare Europe GmbH, Rosselló i Porcel, Barcelona, Spain) containing 200 mL of freshly prepared AT-Sepharose 6B (GE Healthcare Europe GmbH, Rosselló i Porcel, Barcelona, Spain) equilibrated with 0.28 M NaCl and 5 mM Na-phosphate (pH 7.5) buffer. Then, the column was washed with the same buffer until the A_280_ reached values close to 0, and the protein fraction not retained by the AT-Sepharose 6B column was discarded. The retained protein fraction was further eluted with the same buffer containing 0.2 M lactose. Fractions of 10 mL were collected, and those containing proteins were pooled and concentrated with an Amicon system using a Y10 membrane (Millipore Ibérica S.A.U., Madrid, Spain) membrane down to 5 mL. The concentrated protein was applied to a Superdex 75 (26/60; GE) column equilibrated with 400 mM NaCl and 5 mM Na-phosphate (pH 7.5) buffer and was eluted with the same buffer at 2.5 mL/min. The first 70 mL were discarded, and then, fractions of 2.5 mL were taken and their A_280_ measured. Those fractions containing protein were pooled, dialyzed against water and, finally, concentrated as above to reach concentrations of 2.5–4 mg/mL. Finally, the protein solutions were divided in aliquots of 0.1 mL and stored frozen at −20 °C. The purity of the proteins was assessed by SDS-PAGE.

### 4.2. Isolation of Crude Extracts

In some cases, small extract preparations (10 g of material extracted with 100 mL of extraction buffer) of blossoms, early green fruits and early fruit stalks were prepared for comparison of their SDS-PAGE protein profiles. They were obtained just as for the preparation of purified lectins described above, except that they were not subjected to chromatography. After, they were concentrated five-fold by ultracentrifugation with Millipore Amicon ultra 0.5 centrifugal filter units (Ultracel^®^ 10K, Millipore Ibérica SAU, Madrid, Spain) and stored at −20 °C until use.

### 4.3. SDS-Polyacrylamide Gel Electrophoresis of d-Galactose-Binding Lectins from *S. ebulus* Blossoms

The analysis of proteins by SDS-PAGE [31] was performed with the MiniVE (GE Healthcare Europe GmbH, Rosselló i Porcel, Barcelona, Spain) system using 4% stacking gel and 15% polyacrylamide separation slab gels. The protein samples in the loading buffer (62.5 mM Tris-HCl (pH 6.8), 2% (*w*/*v*) SDS, 10% (*v*/*v*) glycerol and 0.025% (*w*/*v*) bromophenol blue) were incubated for 5 min in a boiling water bath. When added, 2-mercaptoethanol (2-ME) was at 3.75%. Twenty microliters of sample buffer containing the protein sample were loaded into each well, and electrophoresis was carried out at 20 °C and 25 mA per gel, using a buffer containing 25 mM Tris-HCl (pH 8.3), 192 mM glycine and 0.1% (*w*/*v*) SDS. Gels were then stained overnight with mild shaking with a solution containing 1% (*w*/*v*) Coomassie Brilliant Blue R-250 (Sigma-Aldrich Química S.L., Tres Cantos, Madrid), 50% (*v*/*v*) methanol and 10% (*v*/*v*) acetic acid, and further, the stain was removed by shaking gels with a solution containing 5% (*v*/*v*) methanol and 7% (*v*/*v*) acetic acid.

### 4.4. Mass-Spectrometry Analysis of the Lectins

Mass spectrometry analysis of protein was carried out as described recently [19]. Briefly, 1 µL of sample (3.8 µg/µL) was spotted onto the sample target plate and allowed to air dry at room temperature. Then, 0.5 µL of a saturated solution of sinapinic acid (Sigma) in 30% acetonitrile and 0.3% trifluoroacetic acid (TFA) were added to the dried protein spots and, again, allowed to air dry at room temperature. Then, analyses in the MALDI-TOF/TOF mass spectrometer operating in positive linear mode (accelerating voltage of 20,000 V) were conducted. An external mass spectrum calibration was performed using BSA as the standard.

### 4.5. Tryptic Fingerprinting and Database Search

Tryptic fingerprinting was performed as described previously [19]. The samples were reduced with 10 mM dithiothreitol (DTT) in 25 mM ammonium bicarbonate for 30 min at 37 °C and subsequently alkylated with 55 mM iodoacetamide in 25 mM ammonium bicarbonate for 15 min in the dark. Finally, samples were digested with 1:20 sequencing-grade trypsin (Roche Molecular Biochemicals, Sant Cugat del Valles, Barcelona, Spain) in 25 mM ammonium bicarbonate (pH 8.5), overnight at 37 °C. After digestion, 1 µL was spotted onto a MALDI target plate and allowed to air dry at room temperature. Then, 0.5 µL of a 3 mg/mL of α-cyano-4-hydroxy-transcinnamic acid matrix in 50% acetonitrile were added to the dried peptide digest spots and, again, allowed to air dry at room temperature. MALDI-TOF MS analyses were performed with a 4800 Proteomics Analyzer MALDI-TOF/TOF mass spectrometer (Applied Biosystems MDS Sciex, Toronto, Canada) operated in positive reflector mode (accelerating voltage of 20,000 V). Mass spectra were obtained (Nd:YAG laser at 355 nm, 40 shots/sub-spectrum for 800 Total shots/spectrum) by reflector positive mode. Mass spectra were internally calibrated using peptides from the auto-digestion of trypsin. Proteins identified ambiguously by peptide mass fingerprinting were subjected to MS/MS sequencing analyses. Thus, from the MS spectra, suitable precursors were selected for MS/MS analyses with CID in (atmospheric gas was used) 1 KV ion reflector mode and precursor mass; windows ±2 Da. For protein identification, the non-redundant NCBI Viridiplantae (Green Plants) database (1,716,557 sequences) was searched using MASCOT 2.3 through the Global Protein Server v3.6 from Applied Biosystems. 

### 4.6. Toxicity of Dwarf Elder Blossom D-Galactose-Specific Lectins

Ebulin blo or SELblo dissolved in water up to the required concentrations adjusted for the mice weights were administered in 60 µL to Swiss mice by nasal instillation under mild isoflurane anesthesia to ease mice handling. Controls with the same volumes of water did not affect survival and body weight. Owing to the clarity of the results, only seven animals per group were used. Treatments, experiments and euthanasia of mice were conducted according to the European Communities Council guidelines (2010/63/EU) concerning the protection of experimental animals. 

## 5. Conclusions

Two lectins, which we named ebulin blo and SELblo, have been found in *S. ebulus* blossoms. Ebulin blo (mass 63,225 *m*/*z*), like the ebulin present in leaves and fruits, are composed of two polypeptide chains of apparent molecular masses of 28,000 (A chain) and 35,000 (B chain) linked by a disulfide bond. SELblo (mass 68,432 *m*/*z*) is devoid of apparent toxicity and has two identical subunits (B chains) of an apparent molecular mass of 36,000 linked by a disulfide bond. Both lectins share amino acid sequence homology with the elderberry allergen, Sam n1. The nasal instillation of a 5 mg/kg body weight of an ebulin blo solution in water triggers lethal toxicity in mice, within five days after the administration. We believe that the administration as an aerosol could be even more toxic than in solution. The toxicity of ebulin blo raises concern due to its potential as a bioweapon, like ricin. More studies should be carried out in order to ascertain the toxicological relevance in that context and to develop analytical procedures for its detection, potential prevention and therapeutics.
